# Porcine Neonatal Blood Dendritic Cells, but Not Monocytes, Are More Responsive to TLRs Stimulation than Their Adult Counterparts

**DOI:** 10.1371/journal.pone.0059629

**Published:** 2013-05-08

**Authors:** Gael Auray, Marina R. Facci, Jill van Kessel, Rachelle Buchanan, Lorne A. Babiuk, Volker Gerdts

**Affiliations:** 1 Vaccine and Infectious Disease Organization, University of Saskatchewan, Saskatoon, Canada; 2 Faculté de Médecine Vétérinaire, Université de Montréal, Saint-Hyacinthe, Canada; 3 Department of Veterinary Microbiology, Western College of Veterinary Medicine, University of Saskatchewan, Saskatoon, Canada; 4 University of Alberta, Edmonton, Canada; Federal University of São Paulo, Brazil

## Abstract

The neonatal immune system is often considered as immature or impaired compared to the adult immune system. This higher susceptibility to infections is partly due to the skewing of the neonatal immune response towards a Th2 response. Activation and maturation of dendritic cells (DCs) play an important role in shaping the immune response, therefore, DCs are a target of choice for the development of efficient and protective vaccine formulations able to redirect the neonatal immune response to a protective Th1 response. As pigs are becoming more important for vaccine development studies due to their similarity to the human immune system, we decided to compare the activation and maturation of a subpopulation of porcine DCs in adult and neonatal pigs following stimulation with different TLR ligands, which are promising candidates for adjuvants in vaccine formulations. Porcine blood derived DCs (BDCs) were directly isolated from blood and consisted of a mix of conventional and plasmacytoid DCs. Following CpG ODN (TLR9 ligand) and imiquimod (TLR7 ligand) stimulation, neonatal BDCs showed higher levels of expression of costimulatory molecules and similar (CpG ODN) or higher (imiquimod) levels of IL-12 compared to adult BDCs. Another interesting feature was that only neonatal BDCs produced IFN-α after TLR7 or TLR9 ligand stimulation. Stimulation with CpG ODN and imiquimod also induced enhanced expression of several chemokines. Moreover, in a mixed leukocyte reaction assay, neonatal BDCs displayed a greater ability to induce lymphoproliferation. These findings suggest that when stimulated via TLR7 or TLR9 porcine DCs display similar if not better response than adult porcine DCs.

## Introduction

Antigen presenting cells (APCs), by sampling antigens and presenting them to immune cells, play a key role in the development of the adaptive immune response. APCs include dendritic cells (DCs), monocyte/macrophages and B cells. APCs are present in peripheral tissues and especially at mucosal surface where they sample antigens. Upon encountering pathogens, they reach a mature state through triggering of their Pattern Recognition Receptors (PRRs) by highly conserved Molecular Associated Molecular Patterns (MAMPs). The maturation of APCs is a complex process leading to the loss of endocytic ability, the up-regulation of costimulatory molecules and MHC class II, the switching in surface expression of chemokine receptors and the production of cytokines [1], [2].

Upon maturation, DCs migrate to lymph nodes to activate T helper (Th) cells and drive the immune response towards a Th1, Th2, Th17 or Treg type of response [3], [4]. This unique ability to activate naive T cells and tailor the immune response makes DCs a target of choice for vaccine formulations. DCs express a broad range of Toll like receptors (TLR), an extensively studied family of PRRs. These receptors can be triggered by safe synthetic agonists mimicking TLR microbial ligands that could be used as adjuvants. In humans, the two main DC subsets, conventional and plasmacytoid DCs (cDCs and pDCs) express different sets of TLRs, with cDCs expressing TLR3 while pDCs express TLR7 and TLR9 [5], [6], while in mice both cDCs and pDCs express TLR9 [7]. We recently showed in our porcine model that porcine monocyte-derived DCs (MoDCs) and blood-derived DCs (BDCs) express different levels of TLRs, with MoDCs expressing higher levels of TLR3 while BDCs expressed more TLR7 and TLR9 [8]. Porcine BDCs consist of cDCs and pDCs, respectively characterized as being CD172^+^, MHC II^+^, CD80/86^+^, CD1^+/−^, CD14^−^, CD4^−^, and CD172^+^, MHC II^+^, CD80/86^+^, CD1^+/−^, CD14^−^, CD4^+^ [9]. In a previous study, using phagocytosis assays, lymphoproliferation assays and morphologic observation, we confirmed that the cells we isolated were DCs, and FACS staining with the specific surface markers confirmed the cells were BDCs [10]. We found that class A CpG ODN (TLR9) were particularly efficient activators of BDCs, inducing a full maturation profile with upregulation of co-stimulatory molecules, switching in chemokine receptor expression, production of the cytokine IL-12p40 and of a broad range of functional chemokines that attracted immune cells [8]. However, very little is known about the activation and maturation of porcine neonatal DCs.

The neonatal immune system is often considered immature because of impaired immune responses to a broad range of pathogens leading to higher susceptibility of newborns to infections. The defects observed in the neonatal immune system are not due to a unique mechanism but are most likely due to a sum of factors, including interfering maternal antibodies [11], persistence of foetal immunoregulatory responses [12], skewing of the immune response toward a Th2 profile [13], impaired APC functions [14], [15], serum inhibition by adenosine [16], or differences in immune cells proportions and numbers [17], [18]. Defects in the development of a strong pro-inflammatory and Th1-type immune response are important in the foetus to avoid any dangerous mother-foetus alloimmune reaction [19] and in the neonate after exposure to new antigens during colonization of the skin or the gastrointestinal tract [12]. Indeed, during the first weeks of life the neonatal immune system will be exposed to self-antigens and common environmental antigens and the onset of a strong immune response would be detrimental. But during infection or after vaccination, it is also important that neonates develop a strong and efficient immune response. Some studies have shown that neonates are able to develop adult-like protective Th1 and cytotoxic immune responses in particular circumstances [20]. It would be beneficial to identify vaccine adjuvants that help overcome the tolerogenic neonatal environment and Th2 bias and allow the development of a long lasting Th1 or mixed protective immune response in neonates. The pig is becoming a model of growing importance for the development of human as well as veterinary vaccines. The study of the porcine neonatal immune system may also allow the dissection of mechanisms leading to differences between human and pig innate immune ontogeny. However, very little is known about the porcine neonatal immune system. Vaccines have to be administered early in life to be fully protective, so adjuvants able to overcome the specificity of the neonatal immune system and induce the development of protective and long lasting immune responses are needed.

In this study we compared the effect of the TLR ligands LPS (TLR4), poly I∶C (TLR3), imiquimod (TLR7), class A and class C CpG ODN (TLR9) on the activation and maturation of adult and neonatal porcine monocytes and BDCs. We did not see any major effect of the different TLR ligands on either adult or neonatal monocytes. In contrast to what we were expecting, imiquimod and CpG ODN not only induced maturation of neonatal BDCs, but the levels of activation observed were more pronounced than those observed with adult BDCs. Only neonatal BDCs increased their surface expression of co-stimulatory molecules following CpG ODN stimulation and produced IL-12p40 after incubation with imiquimod. Despite equal proportions of pDCs, neonatal BDCs, but not adult BDCs, produced the anti-viral cytokine IFN-α. Another unexpected finding was that neonatal BDCs showed a stronger ability to induce lymphocyte proliferation in mixed leukocyte reaction (MLR) assays.

## Materials and Methods

### Animals

Eight-week old (adults) or one-week old (neonates) Dutch Landrace pigs were purchased from a specific pathogen free herd with high health status from Prairie Swine Centre, University of Saskatchewan. All experiments were conducted in accordance with the ethical guidelines of the University of Saskatchewan and the Canadian Council of Animal Care. This specific protocol was approved by the University of Saskatchewan Animal Research Ethics Board (Permit Number: 19940211).

### Isolation of monocytes and blood dendritic cells (BDCs)

Pig blood samples were collected by cardiac vein puncture using 60 ml syringes coated with 7.5% ethylenediaminetetraacetic acid (EDTA) for adult pigs or 10 mL vacutainers coated with EDTA (BD Biosciences, Mountain View, CA) for neonates. PBMCs were isolated using a 60% FICOLL-PAQUE® Plus gradient (GE Healthcare, Uppsala Sweden). Monocytes and BDCs were isolated as described elsewhere [8], [10]. Briefly, after incubation with anti-human CD14 microbeads (Miltenyi Biotec, Auburn, CA), monocytes were either kept for culture or depleted from the PBMCs using the Magnetic Activated Cell Sorting system (MACS®, Miltenyi Biotec). The CD14 negative fraction was first incubated with mouse anti-porcine CD172 antibody (Serotec, Oxford, UK) and then with Rat anti-mouse IgG1 microbeads (Miltenyi Biotec). The CD14^−^ CD172^+^ BDCs were then positively selected using MACS and rested overnight before being used in the different assays. BDCs as well as the CD14^−^ CD172^−^ fraction were used directly after isolation for the MLR assay.

### Monocytes and BDC stimulation

Following overnight culture in RPMI 1640 (GIBCO®-BRL, Burlington ON) supplemented with 10% FBS, 1% HEPES (GIBCO®-BRL), 1% MEM non essential amino acids (GIBCO®-BRL), 1% Antibiotic/Antimycotic (GIBCO®-BRL), 0.5 mM β-Mercaptoethanol (culture media) at 1×10^6^ cells/ml in 12-well plates (Corning, Corning NY), monocytes and BDCs were stimulated with the following TLR ligands: 10 µg/ml poly I∶C (Sigma-Aldrich, Oakville, ON), 1 µg/ml LPS (*E. coli* O55:B5, Sigma-Aldrich), 10 µg/ml imiquimod (InVivogen), 5 µg/ml class A CpG ODN 8954 (ggGGACGACGTCGTGgggggG) and 5 µg/ml class C CpG ODN 2429 (TCGTCGTTTTCGGCGGCCGCCG). The cells were cultured in presence of the TLR agonists for 6 h to study mRNA expression and 24 h for enzyme-linked immunosorbent assay (ELISA) and flow cytometry studies. Control cells were cultured in media for the same time without TLR ligand stimulation. Data were collected from 5 to 6 adult or neonatal pigs and cells were isolated and collected independently on different days.

### Flow cytometry

After a 24 h stimulation, monocytes and BDCs were harvested and resuspended in 1× phosphate buffered saline (PBS) pH 7.3 containing sodium azide (0.03%) and gelatin (0.02%) (staining media) and stained to assess CD80/86 cell surface expression by flow cytometry as described elsewhere [8]. Briefly, cells were incubated 20 min at 4°C with human CD152 (Human CTLA fusion protein IgG2a, Ancell, Bayport, MN). After 3 washes in staining media, cells were incubated for 20 min at 4°C with a fluorescein isothiocyanate (FITC)-conjugated anti-mouse IgG2a monoclonal antibody (mAb) (Southern Biotech, Birmingham, AL) before being fixed in 2% formalin. 10000 events were collected on a FACSCALIBUR™ (BD Biosciences, Mountain View, CA) using the CELLQUEST™ software. The specific CD80/86 staining was calculated by subtracting from the percentage of CD80/86 positive cells, the percentage of non-specific staining obtained by labelling of the cells with an irrelevant mAb of the same isotype (Caltag Laboratories, Burlingame, CA).

### Reverse transcription- quantitative real-time polymerase chain reaction (RT-qPCR)

After 6 h of stimulation with the TLR ligands, BDCs were harvested in TRIZOL® (Invitrogen, Carlsbad, CA) and stored at −80°C. mRNA extraction was performed using Qiagen RNEASY® mini-columns (Qiagen, Mississauga, ON) according to manufacturer's instructions and mRNA quality and quantity was assessed using an Agilent 2100 Bioanalyzer (Agilent Technologies, Mississauga, ON). The SUPERSCRIPT® III PLATINUM® Two-Step RT-qPCR Kit (Invitrogen) was used to generate cDNA according to the manufacturer's instruction. Quantification of cDNA by SYBR green incorporation was performed using a Biorad ICYCLER IQ® (Biorad, Hercules, CA). The sequence of the primers used have already been published elsewhere [8], and the hybridization temperature was 60°C for all of them. To assess the changes in gene expression, we used the comparative cycle threshold (Ct) method [21] where the Ct of the reference gene was subtracted from the Ct of the gene of interest for each sample to calculate the ΔCt. The equation 2^−(ΔCt)^ was used to calculate the relative mRNA levels. To calculate the ΔΔCt, the ΔCt of the control was subtracted from the corresponding stimulated sample and the fold change was determined by the equation 2^−(ΔΔCt)^. Ribosomal protein L (RPL) 19 and Glyceraldehyde 3-phosphate dehydrogenase (GAPDH) were used as reference genes given their stable expression in BDCs as tested with the GeNorm kit [22].

### Enzyme-Linked immuno sorbent assay (ELISA)

1×10^6^ monocytes or BDCs were stimulated 24 h with TLR ligands and the supernatant were collected to determine the cytokine concentrations. The concentrations of IL-6, IL-10, IL-12p40, and TNF-α were determined by using porcine specific R&D Systems DUOSET® commercial kits (R&D Systems, Minneapolis, MN) according to the manufacturer's instruction. ELISA for IFN-α was performed as previously described [23].

### Mixed leukocyte reaction (MLR)

BDCs isolated from adult and neonatal pigs were cultured during 5 days with 5×10^4^ allogeneic CD14^−^ CD172^−^ PBMCs isolated from either adult or neonatal pigs. Cells were cultured in triplicates in 96-well plates with different APC/effector-cell ratios in 200 µl medium. CD14^−^ CD172^−^ PBMCs cultured alone served as negative control and CD14^−^ CD172^−^ PBMCs cultured with 2.5 µg/ml Concanavalin A (ConA) (Sigma) were used as positive controls. 0.4 µCi of [3H] thymidine (Amersham Pharmacia Biotech, Baie de Urfe, PQ) was added during the last 8 h of culture. Cells were then harvested and thymidine incorporation determined using a liquid scintillation counter. No difference in induction of proliferation was seen when BDCs were treated with 50 µg/ml mitomycin C (Sigma), confirming the proliferation observed is due to CD14^−^ CD172^−^ PBMCs and not BDCs.

### Statistical analysis

All the data were statistically analysed using GRAPHPAD PRISM™ 5 software (GraphPad Software, Inc., La Jolla, CA). Differences between groups were assessed using the paired t test (paired, Gaussian distribution), the Wilcoxon signed rank test (paired, non-Gaussian distribution), or the Mann-Whitney U test for non-paired, non-Gaussian data. When P≤0.05, differences were considered significant.

## Results

### Neonatal and adult monocytes stimulation by TLR ligands

**Figure 1:**
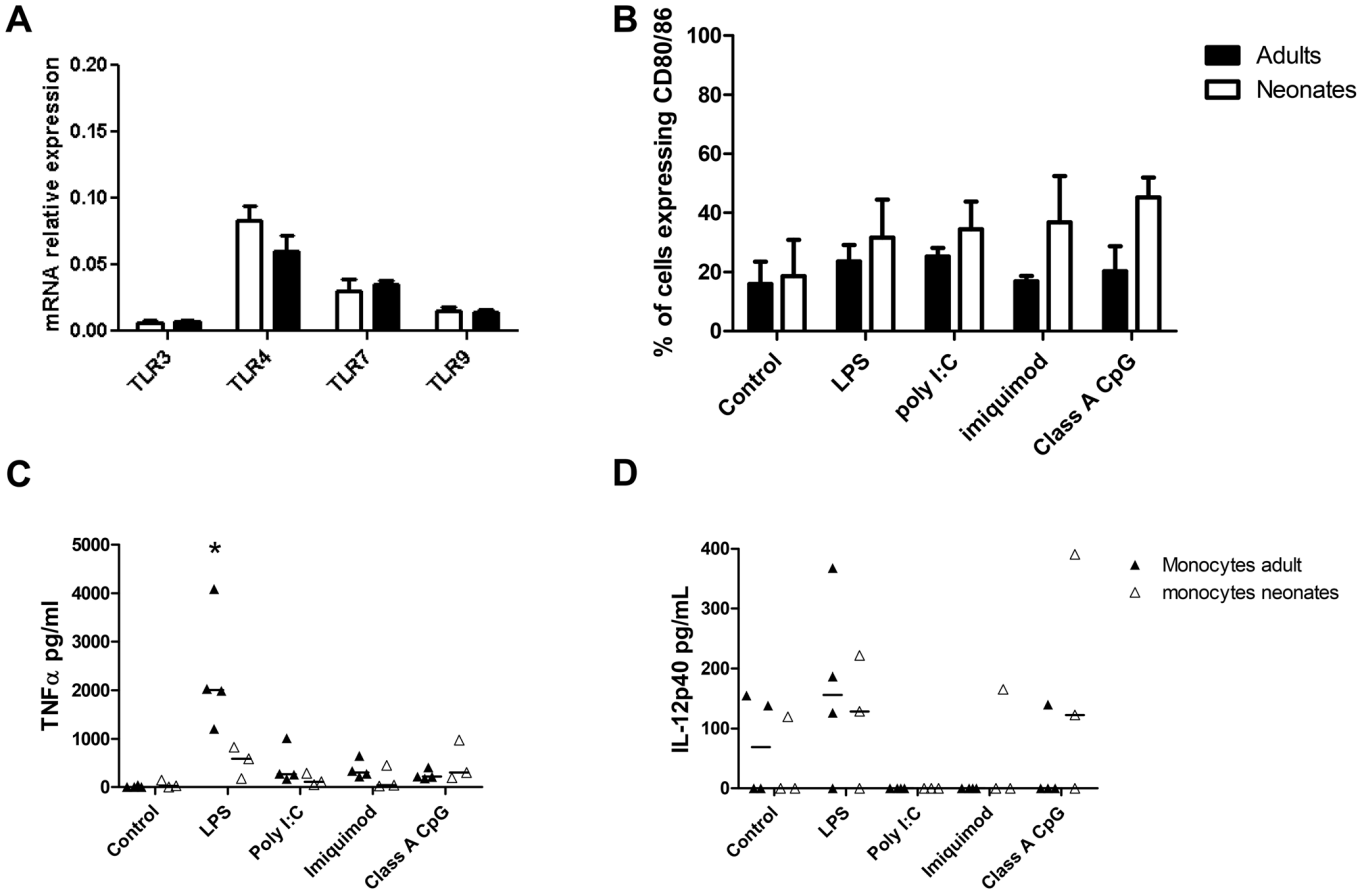
Adult and neonatal monocyte stimulation by TLR ligands. (A) Expression of TLR3, 4, 7 and 9 by non-stimulated adult and neonatal monocytes was assessed using RT-qPCR and is expressed as relative mRNA expression using the 2^−ΔCt^ calculation method. Data represent the mean relative mRNA expression ± SD from 4 different animals. (B) Adult (black boxes) and neonatal (white boxes) monocytes were stimulated 24 h with LPS (1 µg/ml), poly I∶C (10 µg/ml), imiquimod (10 µg/ml), class A or class C CpG ODN (5 µg/ml) or left non-stimulated (control) and were then harvested and prepared for co-stimulatory molecules staining. The percentage of adult and neonatal monocytes expressing CD80/86 was determined using flow cytometry. For each of the TLR stimulation and the corresponding control, data show the mean percentage of positive cells ± SD obtained for cells isolated from 4 animals. (C) and (D) Monocytes were stimulated with TLR ligands 24 h as in (B), then the supernatants were harvested and the concentration of the different cytokines determined by ELISA. We show here the concentration of TNF-α (C) and IL-12p40 (D) in the supernatants from adult (black boxes) or neonatal (white boxes) monocytes. Each data point represents one animal with median value for each group represented by the horizontal bar.

Circulating monocytes represent up to 15% of the peripheral blood mononuclear cells (PBMCs) and are easily sorted by a CD14 magnetic selection. We isolated monocytes from 1-week old neonatal piglets and 8-week old adult pigs, and assessed their TLR expression using RT-qPCR. We did not observe any differences in TLR expression between adult and neonatal monocytes, both populations expressing TLR3, TLR4, TLR7 and TLR9, with TLR4 being the most expressed (Figure 1A). The isolated monocytes were rested overnight and stimulated for 24 hours with TLR ligands. Cells were then harvested to assess their cell surface expression of co-stimulatory molecules by flow cytometry, and the supernatants kept for cytokines measurement. None of the TLR ligands used induced a significant upregulation of co-stimulatory molecules at the surface of adult or neonatal monocytes compared to non stimulated cells, even though imiquimod and class A CpG ODN seemed to induce a slight upregulation in neonates compared to adults (Figure 1B). When supernatants were tested for cytokine production, we found that LPS induced TNF-α production by adult monocytes but not by neonatal monocytes (Figure 1C). No production of TNF-α was seen after any other TLR stimulation either in adult or neonatal monocytes. Basal levels of IL-12p40 were detected in supernatants from stimulated and non-stimulated adult and neonatal cells, but all TLR ligands failed to induce any significant increase in IL-12p40 production following stimulation (Figure 1D). No IL-10 or IFN-α were detected in the supernatants (data not shown). Monocytes isolated from adult or neonatal pigs did not display characteristics of activated cells following stimulation by TLR ligands, so we decided to focus our study on the comparison of adult and neonatal porcine DCs, isolating them directly from circulating blood.

### Conventional and plasmacytoid subpopulations in neonatal and adult BDCs

**Figure 2:**
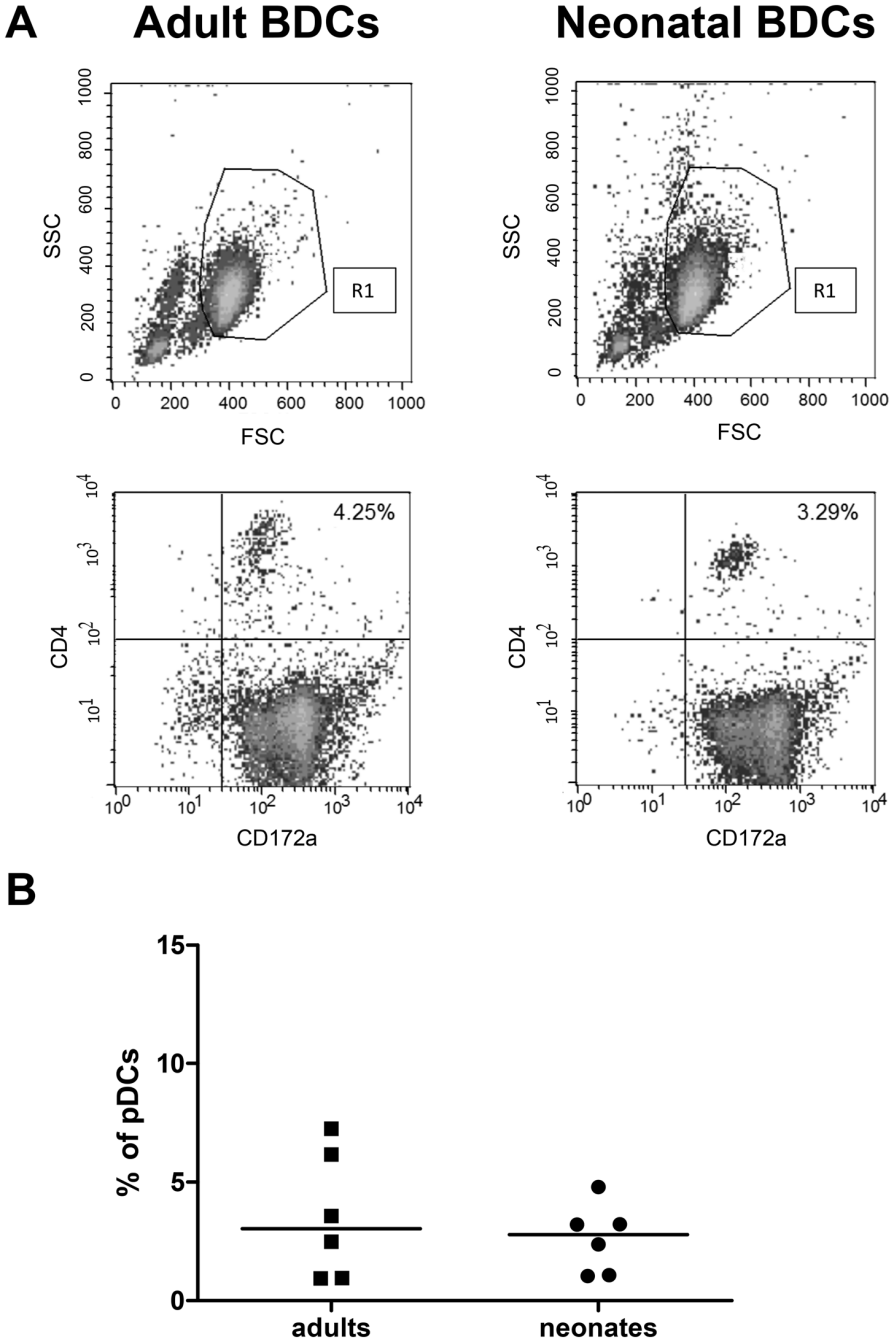
Neonatal and adult porcine BDC populations. (A) Flow cytometry was run on adult (left panel) and neonatal (right panel) BDCs following isolation of the cells. The upper panel represents the size (forward scatter or FSC) and the granulosity of unstained cells (side scatter or SSC), while the bottom panel represents the double staining of adult and neonatal BDCs with CD4 and CD172. (B) BDCs were stained with CD4 and CD172 and the percentage of CD172^+^ CD4^+^ plasmacytoid DCs was determined by flow cytometry. Each data point represents one animal with median value for each group represented by the horizontal bar.

Before studying the activation and maturation of BDCs isolated from blood of adult or neonatal animals, we studied the phenotype of the two populations. Studies in humans [24] and in mice [18] have shown that the proportions of plasmacytoid and conventional DCs can differ between adult and neonates. Using flow cytometry we found that adult and neonatal BDCs populations displayed the same spread and the same gate was used for both populations (Figure 2A, top panels). The purity of the BDCs isolated from neonatal or adult animals was >70% for both adult and neonatal BDCs. The contaminating cells were identified as monocytes. Since monocytes did not display characteristics of mature cells following TLR stimulation, we were able to rule out that the contamination would skew our results (Figure 1). Double staining for CD172a and CD4 confirmed that the BDCs isolated from adult and neonatal blood were constituted of the two expected populations, a CD172a^+^ CD4^−^ population corresponding to the conventional DCs and a CD172a^+^ CD4^+^ population being the plasmacytoid DCs, as previously described [25] (Figure 2A, bottom panels). We did not find any significant differences in the proportion of plasmacytoid DCs in adult compared to neonatal BDCs (3.56±2.65 for adult BDCs and 2.62±1.44 for neonatal BDCs, Figure 2B). Therefore, any differences seen in BDC activation and maturation during this study will likely to be due to differences in intrinsic function of the cells rather than to differences in the proportion of the different DC subpopulations.

### TLR expression by adult and neonatal BDCs

**Figure 3:**
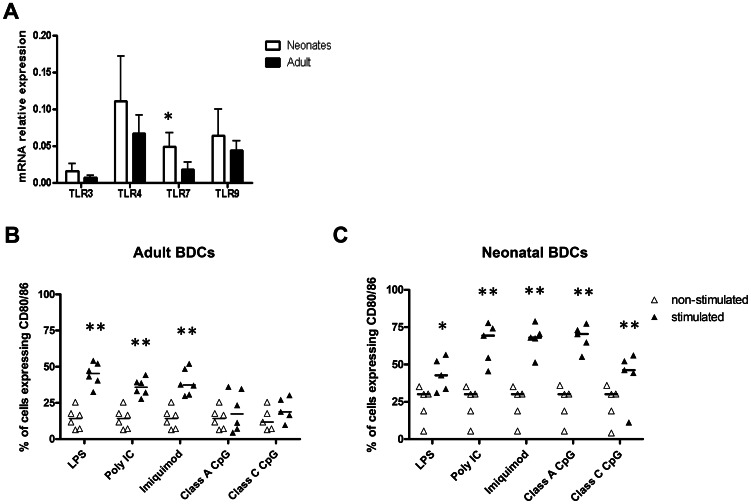
TLRs and co-stimulatory molecules expression by adult and neonatal BDCs. (A) Expression of TLR3, 4, 7 and 9 by non-stimulated adult and neonatal BDCs was assessed using RT-qPCR and is expressed as relative mRNA expression using the 2^−ΔCt^ calculation method. Data represent the mean relative mRNA expression ± SD from 6 different animals. (*p<0.05, Mann-Whitney U test). (B, C) Adult and neonatal BDCs were stimulated (▴) 24 h with LPS (1 µg/ml), poly I∶C (10 µg/ml), imiquimod (10 µg/ml), class A or class C CpG ODN (5 µg/ml) or left non-stimulated (▵). Cells were then harvested and prepared for co-stimulatory molecules staining. The percentage of adult (B) and neonatal (C) BDCs expressing CD80/86 was determined using flow cytometry. Each data point represents one animal with median value for each group represented by the horizontal bar. (*p<0.05, **p<0.01, paired t test).

While a few studies have examined TLRs expression in different adult pig DC subsets like MoDCs or Bone Marrow-derived (BM)DCs [8], [26], to our knowledge, the expression of TLRs in neonatal pig DCs has not been studied. The goal of this study was to compare the activation and maturation levels of adult and neonatal BDCs following TLR ligand stimulation, so we first assessed the level of expression of the relevant TLRs in unstimulated BDCs isolated from adult or neonatal pigs. We found that both neonatal and adult BDCs expressed the same set of TLRs (Figure 3A). While neonatal BDCs displayed similar levels of TLR3, TLR4 and TLR9 compared to adult BDCs, they expressed significantly higher levels of the intracellular receptor TLR7 (Figure 3A). These two DC populations also expressed the same level of TLR5 and TLR8 mRNA (Data not showed).

### Cell surface expression of co-stimulatory molecules following TLR ligand stimulation

Knowing that BDCs isolated from pig adults or neonates expressed the relevant TLRs, we compared the activation and maturation of these cells following stimulation with the corresponding TLR ligand: poly I∶C (TLR3), LPS (TLR4), imiquimod (TLR7), class A and class C CpG (TLR9). Some of the most commonly used activation markers are the expression of co-stimulatory molecules like CD80 and CD86 on the surface of the DCs, which can induce T cells activation through CD28 triggering. Following 24 h of stimulation with these TLR ligands, we stained the cells to assess their expression of CD80/86 by flow cytometry. Stimulation with LPS, poly I∶C and imiquimod significantly increased the surface expression of co-stimulatory molecules in both adult and neonatal BDCs (Figure 3B, 3C). Despite a similar level of expression of TLR9 (Figure 3A), adult and neonatal BDCs did not equally respond to CpG-ODN stimulation. In adult BDCs, class A and class C CpG-ODN stimulation did not induce any increase in cell surface expression of co-stimulatory molecules (Figure 3B). Surprisingly, both class A and class C CpG-ODN stimulation significantly increased the expression of CD80/86 by neonatal BDCs (Figure 3C).

### Chemokine receptors (CCR) expression by adult and neonatal BDCs

**Figure 4:**
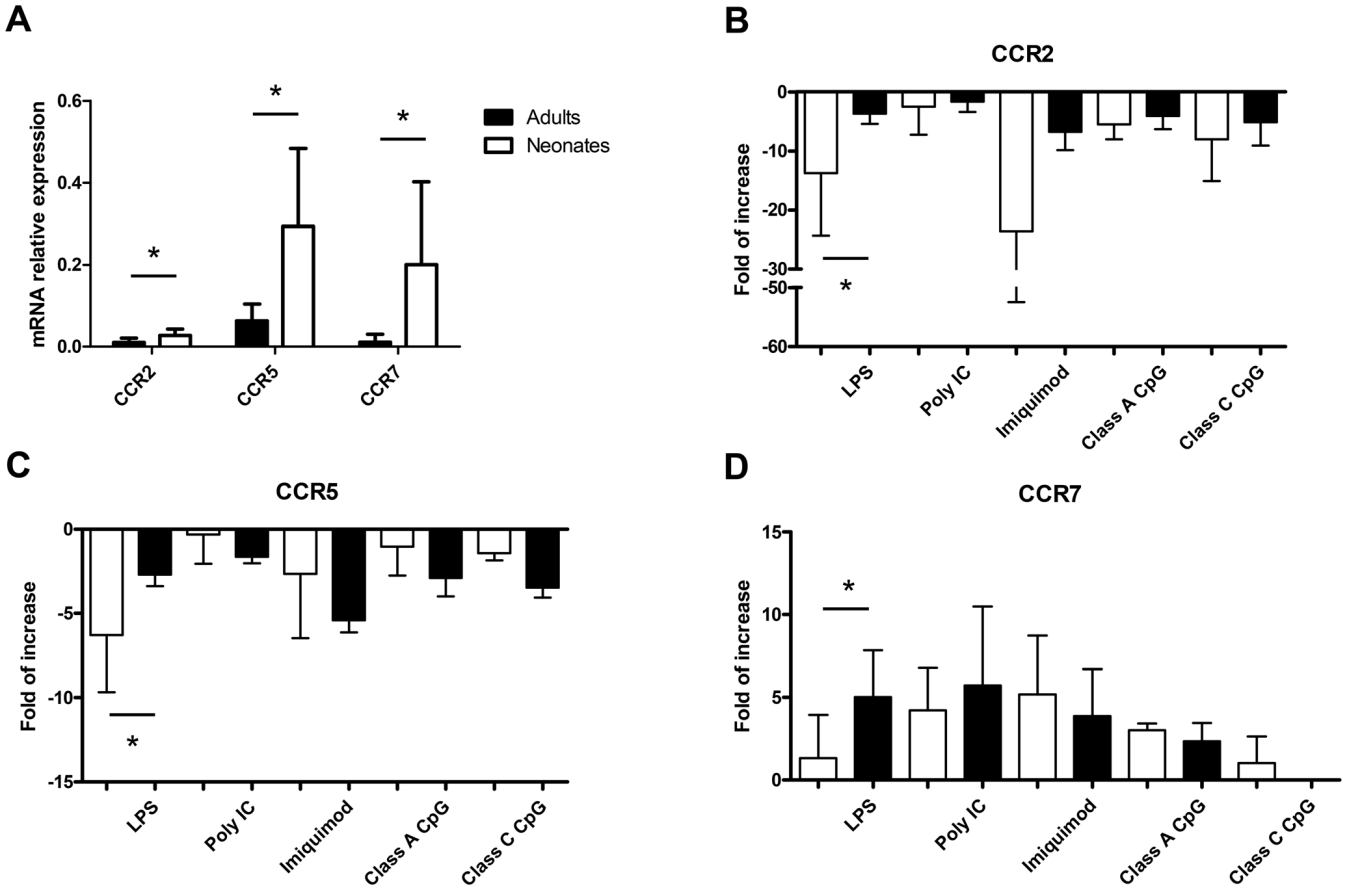
Chemokine receptors expression by adult and neonatal BDCs following TLR stimulation. (A) Expression of CCR2, 5 and 7 by non-stimulated adult and neonatal BDCs was assessed using RT-qPCR and is expressed as relative mRNA expression using the 2^−ΔCt^ calculation method. Data represent the mean relative mRNA expression ± SD from 5 different animals. (*p<0.05, Mann-Whitney U test). (B, C, D) Adult and neonatal BDCs were stimulated 6 h with LPS (1 µg/ml), poly I∶C (10 µg/ml), imiquimod (10 µg/ml), class A or class C CpG ODN (5 µg/ml) or left non-stimulated and then harvested in TRIZOL®. After mRNA extraction, the expression of CCR2 (B), CCR5 (C) and CCR7 (D) was assessed by RT-qPCR. Results show the fold of increase following stimulation compared to the non-stimulated cells using the 2^−ΔΔCt^ and show the mean ± SD from 5 animals. (*p<0.05 Mann-Whitney U test).

Another important mechanism in DCs maturation is the switch in chemokine receptors. Immature DCs express CCR1, −2 and −5, and localize to mucosal surfaces in response to chemokines recognized by these receptors. Upon maturation, these receptors are downregulated and DCs express CCR7 allowing them to respond to CCL19 and CCL21 and migrate to lymph nodes to initiate the adaptive immune response [1]. We first assessed the basal level of chemokine receptors CCR2, CCR5 and CCR7 expression in freshly isolated adult and neonatal BDCs. Neonatal BDCs expressed significantly higher levels of the three chemokine receptors studied (Figure 4A). In both adult and neonatal cells, CCR5 was the most highly expressed chemokine receptor and CCR2 the less expressed. To our surprise, neonatal BDCs expressed levels of CCR7 close to those found for CCR5. This is unusual for immature or non-stimulated DCs that usually express low levels of CCR7 as seen in adult BDCs. We then studied changes in mRNA chemokine receptor expression following 6 h stimulation with TLR ligands using RT-qPCR. Despite a low basal expression of CCR2, LPS, imiquimod, class A and class C CpG-ODN induced a downregulation of chemokine receptor expression in both adult and neonatal BDCs (Figure 4B). This downregulation was significantly more important in neonatal BDCs following LPS stimulation, whereas the decrease in CCR2 expression was comparable in adult and neonatal cells following imiquimod, class A or class C CpG-ODN treatments. The same pattern of expression was observed for CCR5 with no significant differences between adults and neonates seen following imiquimod, class A or class C CpG-ODN stimulation (Figure 4C). As seen for CCR2, poly I∶C did not induce any downregulation of the expression of CCR5 in adult or neonatal BDCs, and LPS induced a significantly higher downregulation of CCR5 in neonatal BDCs (Figure 4C). In both adult and neonatal BDCs, imiquimod induced the expression of CCR7 and, whereas it had no effect on the expression of CCR2 and CCR5, poly I∶C induced a comparable increase in the expression of this chemokine receptor involved in the homing of mature DCs to the lymph nodes (Figure 4D). Despite a greater downregulation of CCR2 and CCR5 in neonatal BDCs following LPS stimulation, the TLR4 ligand failed to induce the expression of CCR7 compared to adult BDCs (Figure 4D)

### Induction of chemokines by TLR ligands stimulation in adult and neonatal BDCs

**Figure 5:**
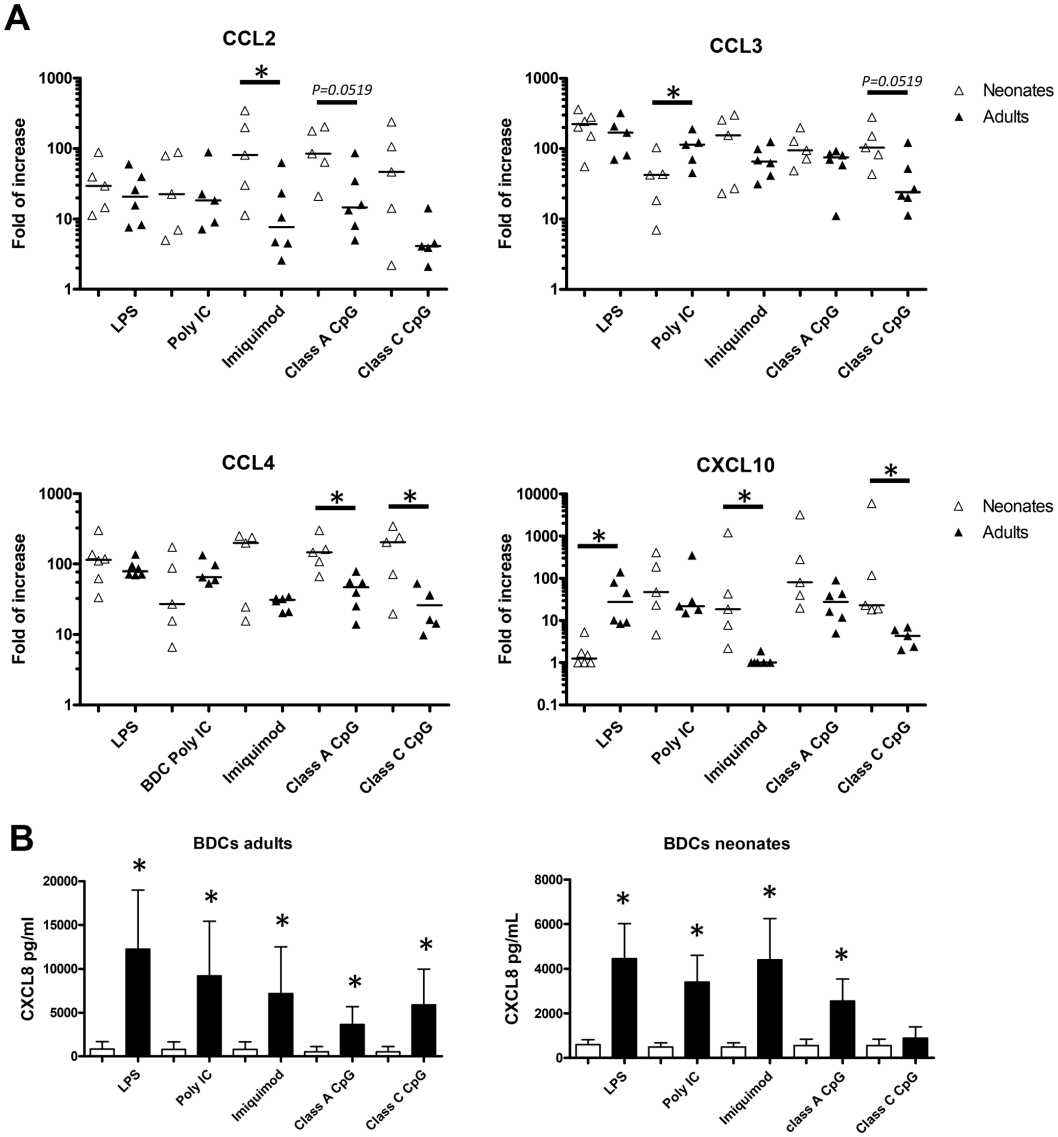
Chemokines expression and production by stimulated adult and neonatal BDCs. Porcine BDCs from 8-week old adults and 1-week old neonates were cultured 6 h with LPS (1 µg/ml), poly I∶C (10 µg/ml), imiquimod (10 µg/ml), class A or class C CpG ODN (5 µg/ml) and were then harvested in TRIzol. After mRNA extraction, the gene expression of CCL2, CCL3, CCL4 and CXCL10 by stimulated adult (▴) and neonatal (▵) BDCs was determined. Each of the data point corresponds to one animal and displays the fold of increase compared to unstimulated cells with the median, using the 2^−ΔΔCt^ calculation method. (*p<0.05 Mann-Whitney U test). (B) Adult and neonatal BDCs were stimulated 24 h with the different TLR ligands and the supernatants were harvested. The concentration of CXCL8 was assessed by ELISA, showing the production of the chemokine after stimulation (black boxes) or without stimulation (white boxes). Results show the mean production of CXCL8 in pg/ml ± SD from 5 or 6 animals. (*p<0.05, Wilcoxon signed rank test).

Because of their location in peripheral tissues and at mucosal surfaces DCs are amongst the first cells present at the site of infection and have an important role in triggering the immune response and amplify the recruitment of immune cells by producing chemokines. This chemokine production by activated DCs has been studied in mice [27], [28] and humans [29]–[31], and we recently showed that in pigs, adult BDCs stimulated by TLR ligands increased their expression of a broad range of chemokines [8]. We compared the chemokine mRNA expression (6 h stimulation) or protein production (24 h stimulation) by adult and neonatal BDCs stimulated with TLR ligands. The expression of several CC-(CCL2, CCL3, CCL4, CCL20) and CXC-(CXCL2 and CXCL10) chemokines and the production of CXCL-8 were studied. No differences were seen for CCL20 and CXCL2, with all the TLR ligands inducing a comparable upregulation in both adult and neonatal BDCs (data not shown). Imiquimod and CpG-ODN stimulations induced significantly higher levels of expression of chemokines by neonatal BDCs (Figure 5A). While imiquimod induced higher levels of the monocyte attracting chemokine CCL2 and the mature T cells attracting CXCL10, CCL4 and CXCL10 were more upregulated following CpG-ODNs stimulation in neonatal BDCs. Even though it was not statistically significant, there was a trend for Class A CpG and class C CpG to induce higher levels of mRNA expression of CCL2 and CCL3 respectively in neonatal BDCs (Figure 5A). Otherwise, neonatal BDCs showed a comparable upregulation of chemokine expression following TLR ligand stimulation compared to adult BDCs, except following poly I∶C stimulation for CCL3 and LPS stimulation for CXCL10, with adult BDCs showing a significantly greater increase in gene expression (Figure 5A). We also assessed by ELISA the production of CXCL8, a chemokine involved in the recruitment of neutrophils. While all the stimulations induced production of CXCL8 by adult BDCs, neonatal BDCs failed to produce the chemokine following class C CpG-ODN (Figure 5B). Although LPS, poly I∶C, imiquimod and class A CpG-ODN induced the production of CXCL8 by neonatal BDCs, the levels of protein found in the supernatants were lower than what was found for adult BDCs (Figure 5B).

### Cytokines production by stimulated adult and neonatal BDCs

**Figure 6:**
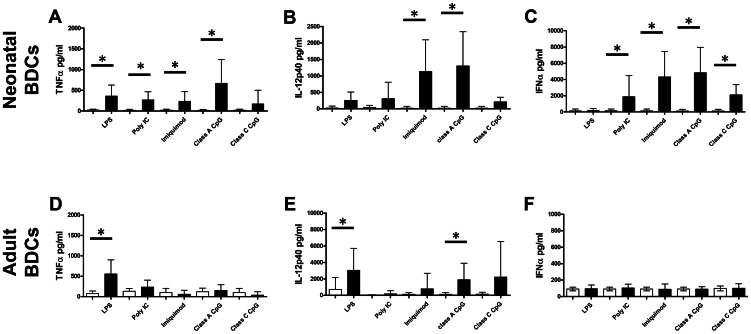
Cytokine production by activated adult and neonatal BDCs. BDCs were stimulated 24 h with LPS (1 µg/ml), poly I∶C (10 µg/ml), imiquimod (10 µg/ml), class A or class C CpG ODN (5 µg/ml), then the supernatants were harvested and the concentration of the different cytokines determined by ELISA. The upper panel shows the results for neonatal BDCs and the bottom panel the ones for adult BDCs. We show here the concentration of TNF-α (A, D), IL-12p40 (B, E) and IFN-α (C, F) in the supernatants from stimulated (black boxes) or non-stimulated (white boxes) BDCs. Results show the mean cytokine production in pg/ml ± SD from 5 or 6 animals. (*p<0.05, Wilcoxon signed rank test).

The higher sensitivity to infection in early life has been linked to a skew in the neonatal immune response towards a Th2 response rather than a protective Th1 immune response. It has been shown in mice and humans that this shift could be partly due to intrinsic defect of cytokines production by APCs, especially DCs that are important in directing the immune response [14]. We compared by ELISA the cytokine production in supernatants of adult and neonatal BDCs after 24 h stimulations with different TLR ligands. LPS induced the production of comparable levels of the pro-inflammatory cytokine TNF-α in adult and neonatal cells (Figure 6A and 6D). TNF-α production by neonatal BDCs was also induced by poly I∶C, imiquimod and class A CpG-ODN, but not when adult BDCs were stimulated. The production of TNF-α observed following LPS stimulation could be due to contaminating population of monocytes, since we observed TNF-α production by monocytes following LPS stimulation (Figure 1C). A different pattern of IL-12p40 production was observed between adult and neonatal cells. While both adult and neonatal BDCs produced IL-12p40 following class A CpG-ODN stimulation, this cytokine was produced only by adult BDCs following LPS stimulation, and only by neonatal BDCs following imiquimod stimulation (Figure 6B and 6E). BDCs include two DCs populations, namely cDCs and pDCs, the latter of which is known for high production of type I IFN following activation. Adult BDCs failed to produce IFN-α following TLR ligand stimulation (Figure 6F). Despite similar proportions of pDCs within the BDC population (Figure 2B), neonatal BDCs, unlike their adult counterparts, produced IFN-α after poly I∶C, imiquimod, class A or class C CpG stimulation, imiquimod and class A CpG inducing the highest levels of this cytokine (Figure 6C). We also studied the production of the anti-inflammatory cytokine IL-10 and the pro-inflammatory cytokine IL-6 following TLR ligands stimulation and both in adult and neonates, no production of these two cytokines was detected in the supernatants of control or stimulated BDCs (data not shown).

### Lymphocyte stimulation by adult and neonatal BDCs

**Figure 7:**
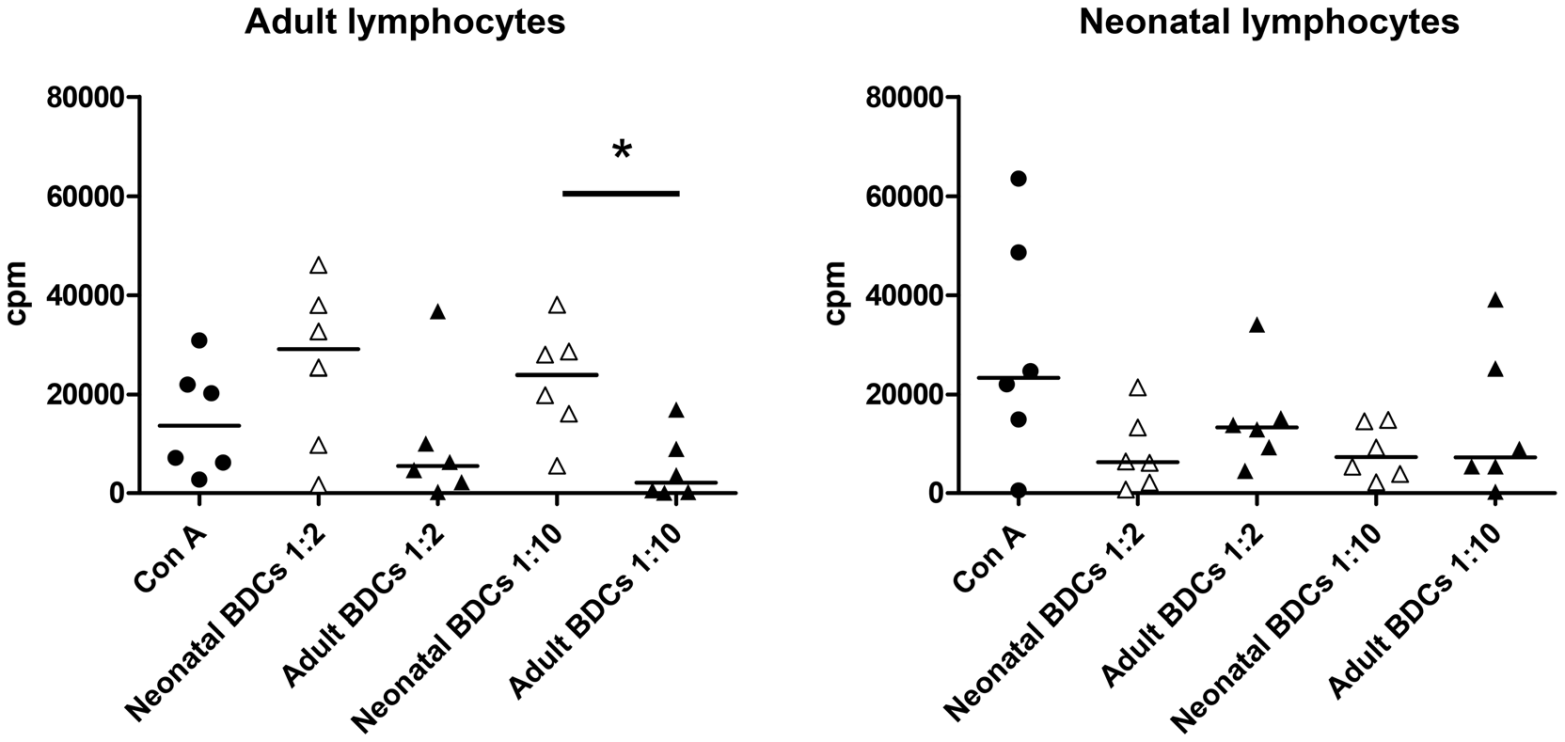
Adult and neonatal lymphocytes mixed leukocyte reaction (MLR) assay. BDCs isolated from adult and neonatal pigs were cultured during 5 days with allogeneic CD14^−^ CD172^−^ PBMCs isolated from either adult (A) or neonatal (B) pigs. The neonatal (white boxes) and adult (black boxes) BDCs were cultured with the CD14^−^ CD172^−^ at two different ratios (1∶2 and 1∶10) and the lymphoproliferation was assessed by tritiated thymidine incorporation using a liquid scintillation counter. Each dot represents cells coming from one animal (n = 6 animals) and the horizontal bar represents the median. (*p<0.05 Mann-Whitney U test).

After finding that adult and neonatal BDCs had different profiles of activation and maturation in response to TLR stimulation, neonatal cells surprisingly displaying higher expression and production of activation markers, we wanted to assess if this translated into differences in lymphocyte stimulation by DCs. For this purpose we decided to assess the ability of adult and neonatal BDCs to induce lymphocyte proliferation in mixed leukocyte reaction (MLR) assays. The principle of this assay is to culture APCs from one animal with lymphocytes from another animal with a different genetic background, and measure the lymphoproliferation after 5 days of co-culture to assess the ability of APCs to stimulate lymphocytes. We used APCs from adults and neonates as well as lymphocytes isolated from adults (Figure 7A) and neonates (Figure 7B). ConA was used as a positive control for lymphocyte proliferation and the cells were co-cultured at DCs to lymphocytes ratios of 1∶2 and 1∶10. At a 1∶2 ratio, no significant differences were seen in induction of adult lymphocyte proliferation by adult or neonatal BDCs, even though the median value for neonatal BDCs is more than 5 times higher than for adult BDCs (29005 cpm and 5506 cpm respectively, Figure 7A). At a 1∶10 ratio, we observed that neonatal BDCs induced a significantly higher proliferation of adult lymphocytes than adult BDCs. When the BDCs were co-cultured with lymphocytes isolated from porcine neonates, no differences were seen in lymphocyte proliferation between neonatal BDCs and adult BDCs for the 1∶2 or 1∶10 ratios (Figure 7B). While the levels of proliferation induced in neonatal lymphocytes by adult BDCs were comparable to those induced in adult lymphocytes, neonatal BDCs lost their ability to induce greater lymphoproliferation when they were co-cultured with neonatal lymphocytes.

## Discussion

It is important to develop neonatal models that will allow the development and the study of vaccine formulations and their efficacy in a neonatal environment. The neonatal immune system has been studied mostly in mouse models that have shown some limitations with results that can be difficult to transpose to humans. Studies in human have also shown limitation in terms of blood and tissue availability and *in vivo* or *ex vivo* experiments, often using human cord blood cells that are more reflecting the fetal immune system rather than the neonatal immune system. Therefore, the pig model has emerged as a promising alternative with a size, a physiology and a course of disease development that are closer to humans [32].

TLR ligands are potent innate immune system activators and strong potential adjuvant candidates for vaccine formulations. APCs, key cells in the presentation of antigen and development of a protective immune response, express a broad range of TLRs and are important targets for these adjuvants. Some of these TLR ligands have been shown to be efficient adjuvants in a neonatal porcine model. The TLR3 ligand poly I∶C induced type I IFN responses to the transmissible gastroenteritis virus (TGEV) [33], while the TLR9 ligand CpG-ODN was shown to induce Th1 immune responses in piglets when used in PRRSV [34], swine streptococcic septicemia killed (SSSK) [35], or pseudorabies attenuated virus (PRV) [36] vaccine formulations. Being easily isolated from blood with a positive magnetic selection for CD14 [10], we first compared the activation and maturation states of adult and neonatal porcine monocytes following stimulation with TLR ligands. Similar to what had been previously observed in swine [37] or humans [38], adult monocytes stimulated with LPS produced more TNF-α than neonatal monocytes. Contrary to the study with human monocytes, we did not observe any difference between adult and neonatal porcine monocytes in terms of TNF-α production following imiquimod stimulation. Because of the lack of effects of TLR ligands on porcine monocytes, we decided to investigate the activation and maturation profiles of adult and neonatal porcine DCs.

Few studies have investigated porcine neonatal DCs, mostly *in vivo* in gnotobiotic piglets [39], [40], but this is to our knowledge the first comprehensive study assessing the activation and maturation profile of isolated porcine neonatal DCs. In mice spleens, differences have been found in DC subpopulations proportions: pDCs were more represented than cDCs and adult-like pDCs to cDCs ratio were reached after 5 weeks [18], [41]. Similar to what we found in our porcine model after isolation of BDCs (Figure 1B), another study in humans showed no differences between adult and neonatal pDC to cDC ratios [42], whereas Jamin *et al.* found that the pDC to cDC ratio in porcine adult blood was 1∶1 [43]. These discrepancies could be due to the different methods used to assess these proportions (whole blood studies versus isolated cells), the PBMCs or DCs isolation protocols (magnetic sorting versus adherence), or the use of frozen cells, whereas in our study we used freshly isolated cells. In our model, any difference seen between adult and neonatal BDCs is therefore not likely to be due to differences between cDCs and pDCs proportions.

In this study, we found that neonatal BDCs displayed a more mature phenotype than adult BDCs after stimulation with TLR7 (imiquimod) and TLR9 ligands (class A and class C CpG-ODN) and to a lesser extent TLR3 ligand poly I∶C, and were also more efficient in inducing lymphocytes proliferation. One of the most important features of DCs maturation is the upregulation of co-stimulatory molecules that gives the second signal needed for T cells activation and proliferation. While some studies using DCs isolated from human cord blood or derived from cord blood monocytes have shown that neonatal DCs display some defects in their co-stimulatory molecules expression compared to their adult counterparts after stimulation with LPS [44] , poly I∶C [44], CpG ODN [42], or the TLR7 ligand Resiquimod [45], other works using neonatal mouse splenic DCs or human cord blood DCs have shown similar co-stimulatory molecules upregulation after LPS [46] or CpG ODN [47], [48]. Unlike what was observed in mice and humans, in our model, TLR3, TLR7 and TLR9 stimulations induced higher levels of co-stimulatory molecules in neonatal BDCs compared to adult BDCs. CpG ODNs, which had no effect in adult BDCs, were potent inducers of co-stimulatory molecules surface expression in neonatal BDCs. The differences seen between these studies could be a result of different DC populations (MoDCs, spleen DCs, cord blood DCs) as well as how they are stimulated (isolated DCs or whole blood or PBMCs stimulation). The neonatal immune system is quickly evolving and the age of the neonates may also be of importance [18], [41], [49]. For example neonatal mice are more immature than human neonates and human cord blood cells may be more representative of the fetal immune system with different immune cells functions for cells from the fetal or adult waves of hematopoietic precursors [50].

Besides the activation of immune cells, efficient vaccine formulations also play an important role in the recruitment of immune cells to the site of injection by inducing the production of chemokines by target cells which triggers an amplification loop leading to a more efficient immune response. We previously showed that following TLR ligand stimulation, porcine BDCs were able to produce a broad range of CC- and CXC-chemokines [8]. We found in this study that neonatal BDCs also had a greater ability to express chemokines after stimulation by TLR7 and TLR9 ligands, especially two important chemokines: CCL2 and CXCL10. CCL2 recruits a subset of circulating monocytes expressing CCR2 which differentiate into inflammatory DCs when they reach the tissues [51], and CXCL10 is involved in the recruitment of CXCR3 expressing activated Th1 and cytotoxic T cells [52], [53]. In the lymph nodes, DCs producing CXCL10 preferentially recruit IFN-γ producing T cells and help the development of a Th1 response by keeping these cells in DC-T cells clusters [28].

One of the difficulties encountered with the development of vaccines for neonates is the development of Th1 immune responses in an environment prone to Th2 or tolerance responses. If Th1 cells can be specifically induced in neonates after primary exposure with an antigen [54], these cells express an IL-4Rα/IL-13Rα1 heteroreceptor that once triggered by IL-4 induces apoptosis of Th1 cells when triggered upon restimulation with the same antigen, thus leading to the development of a Th2 immune response [55]. Neonatal Th1 cells can be protected against IL-4-induced apoptosis through the down-regulation of IL-13Rα1 induced by DCs IL-12 production [56], so using vaccine formulations containing adjuvants inducing IL-12 production by neonatal DCs should be beneficial to neonates and induce protective immunity. We showed here that CpG ODN induced IL-12p40 production by neonatal BDCs to levels comparable to the adult, while imiquimod induced its production only in neonatal BDCs. This production of IL-12p40 was associated with production of TNF-α and IFN-α, and to our surprise adult BDCs did not produce any of those cytokines following TLR7 and 9 stimulations. IFN-α is a key cytokine in the control of viral infections which can drive Th1 and cytotoxic immune responses, activate DCs and enhance cross presentation or primary antibody responses [57]. This unexpected enhanced capacity of neonatal DCs to produce Th1 type cytokines following TLR stimulation has already been reported in mice, goats and sheep and seems to be a common feature of neonatal DCs. In mice, Sun *et al.* showed that CpG ODN or poly I∶C-stimulated splenic DCs produced more IL-12 and IFN-α than adult DCs [41]. Another group showed that stimulation with poly I∶C or resiquimod in goats, and resiquimod or CpG ODN in sheep, induced higher production of both IL-12 and IFN-γ by neonatal MLN cells compared to adults and that cells with similar morphology with DCs were responsible for the IL-12 production [58]–[60].

In summary, we found that TLR7 and TLR9 ligands and to a lesser extent TLR3 ligands did not only induce the activation and maturation of porcine neonatal BDCs, but that the level of activation of these cells was greater than what we observed with adult BDCs. The neonatal BDCs were also more efficient than their adult counterparts in producing Th1 cytokines after imiquimod and CpG ODN stimulation, the most striking being the high levels of the anti-viral and pro-Th1 cytokine IFN-α produced by the neonatal cells while none was detected in adult cells. This more mature phenotype observed in neonatal BDCs was associated to another unexpected result which showed neonatal BDCs more efficient in inducing adult T cells proliferation than adult BDCs, ruling out the hypothesis of any intrinsic deficiency of neonatal DCs. Imiquimod, CpG ODN and also poly I∶C are therefore very promising adjuvants for future neonatal vaccines, especially for vaccines against infections that need a Th1 or a balanced Th1/Th2 immune response.
